# Comparison between estimated and brachial‐ankle pulse wave velocity for cardiovascular and overall mortality prediction

**DOI:** 10.1111/jch.14124

**Published:** 2020-12-12

**Authors:** Po‐Chao Hsu, Wen‐Hsien Lee, Wei‐Chung Tsai, Ying‐Chih Chen, Chun‐Yuan Chu, Hsueh‐Wei Yen, Tsung‐Hsien Lin, Wen‐Chol Voon, Wen‐Ter Lai, Sheng‐Hsiung Sheu, Ho‐Ming Su, Cheng‐An Chiu

**Affiliations:** ^1^ Division of Cardiology Department of Internal Medicine Kaohsiung Medical University Hospital Kaohsiung Taiwan; ^2^ Faculty of Medicine College of Medicine Kaohsiung Medical University Kaohsiung Taiwan; ^3^ Department of Internal Medicine Kaohsiung Municipal Siaogang Hospital Kaohsiung Taiwan

**Keywords:** brachial‐ankle pulse wave velocity, estimated pulse wave velocity, mortality

## Abstract

Pulse wave velocity (PWV) was a good marker of arterial stiffness and could predict cardiovascular (CV) outcomes. Recently, estimated PWV (ePWV) calculated by equations using age and mean blood pressure was reported to be an independent predictor of major CV events. However, there was no study comparing ePWV with brachial‐ankle PWV (baPWV) for CV and overall mortality prediction. We included 881 patients arranged for echocardiographic examination. BaPWV and blood pressures were measured by ankle‐brachial index‐form device. The median follow‐up period to mortality was 94 months. Mortality events were documented during the follow‐up period, including CV mortality (*n* = 66) and overall mortality (*n* = 184). Both of ePWV and baPWV were associated with increased CV and overall mortality after the multivariable analysis. ePWV had better predictive value than Framingham risk score (FRS) for CV and overall mortality prediction, but baPWV did not. In direct comparison of multivariable analysis using FRS as basic model, ePWV had a superior additive predictive value for CV mortality than baPWV (*p* = .030), but similar predictive valve for overall mortality as baPWV (*p* = .540). In conclusion, both ePWV and baPWV were independent predictors for long‐term CV and overall mortality in univariable and multivariable analysis. Besides, ePWV had a better additive predictive value for CV mortality than baPWV and similar predictive value for overall mortality as baPWV. Therefore, ePWV obtained without equipment deserved to be calculated for overall mortality prediction and better CV survival prediction.

## 1 INTRODUCTION

Arterial stiffness measured by pulse wave velocity (PWV) is associated with micro‐ and macro‐vascular complications, and it can also predict cardiovascular (CV) outcomes and mortality in the literature.^1^, ^2^, ^3^, ^4^, ^5^, ^6^, ^7^, ^8^, ^9^ Although there are several methods to measure PWV, brachial‐ankle PWV (baPWV) was considered as a good index of arterial stiffness and exhibited similar extents of associations with CV disease risk factors and clinical outcomes with carotid‐femoral PWV, which was the most recognized and established index of arterial stiffness.^10^ Recently, estimated PWV (ePWV) calculated by equations using age and mean blood pressure (MBP) was also reported to be an independent predictor of major CV events.^11^, ^12^, ^13^, ^14^ However, there was no literature comparing ePWV with baPWV for long‐term CV and overall mortality prediction. Hence, the present study was designed to examine the ability of ePWV in prediction of long‐term CV and all‐cause mortality and compare the predictive value of long‐term CV and all‐cause mortality between ePWV and baPWV.

## 2 METHODS

### 2.1 Study population and design

Study subjects were randomly included from a group of patients who were arranged for echocardiographic examinations at Kaohsiung Municipal Siaogang Hospital from March 2010 to March 2012 due to ischemic heart disease, heart failure, hypertension, abnormal cardiac physical examination, survey for dyspnea, and the pre‐operative cardiac function survey. Patients with significant mitral or aortic valve diseases, atrial fibrillation, inadequate image visualization, or ankle‐brachial index (ABI < 0.9) were excluded. The reason why we excluded ABI < 0.9 was due to unreliable measurement of PWV under the situation of peripheral artery stenosis or occlusion.^15^, ^16^ Finally, 881 patients were enrolled in this study.

### 2.2 Ethics statement

The study protocol was approved by the institutional review board (IRB) committee of our Hospital. Informed consents have obtained in written form from patients and all clinical investigation was conducted according to the principles expressed in the Declaration of Helsinki.

### 2.3 Assessment of baPWV and ePWV

Around 10 min after the completion of echocardiographic examination, baPWV was evaluated using an ankle‐brachial index‐form device (VP1000; Colin, Aichi, Japan), which automatically and simultaneously measures blood pressures in bilateral arms and ankles by an oscillometric method.^17^, ^18^ For measuring baPWV, pulse waves that were acquired from the brachial and tibial arteries were recorded simultaneously, and the transmission time, which was defined as the time interval between the initial increase in brachial and tibial waveforms, was determined. The transmission distance from the arm to each ankle was calculated according to body height. The value of baPWV was automatically calculated as the transmission distance divided by the transmission time. After obtaining bilateral baPWV values, the higher value was used for later analysis.

For calculation of ePWV, we used the equation described in the study by Greve et al that was derived by the Reference Values for Arterial Stiffness' Collaboration.^11^, ^14^ The ePWV was calculated from age and MBP: ePWV = 9.587−0.402 × age + 4.560 × 10^−3^ × age^2^−2.621 × 10^−5^ × age^2^ × MBP + 3.176 × 10^−3^ × age × MBP−1.832 × 10^−2^ × MBP. MBP was calculated as diastolic blood pressure + 0.4(systolic blood pressure – diastolic blood pressure). After obtaining bilateral ePWV values, the higher value was used for later analysis.

### 2.4 Collection of demographic and medical data

Demographic and medical data including age, gender, smoking status, and comorbidities were obtained from medical records or interviews with patients. In addition, information about patient medications including aspirin, angiotensin‐converting enzyme inhibitors, angiotensin II receptor blockers, β‐blockers, calcium channel blockers, and diuretics at enrollment was obtained from medical records.

### 2.5 Calculation of Framingham risk score (FRS)

Framingham risk score was used as the basic model to further compare the predictive value of ePWV and baPWV in multivariable analysis. FRS was calculated by a computer program and based on using a previously reported algorithm which includes the parameters of age, sex, total cholesterol, HDL cholesterol, systolic blood pressure, smoking, presence of diabetes, and being under treatment for hypertension.^19^

### 2.6 Definition of CV and all‐cause mortality

All study patients were followed up till December 2018. Information of survival and causes of death were obtained from the official death certificate and final confirmation by the Ministry of Health and Welfare.

### 2.7 Statistical analysis

SPSS 22.0 software (SPSS, Chicago, IL, USA) was used for statistical analysis. Data were expressed as mean ± standard deviation, percentage, or median (25th–75th percentile) for follow‐up period. Continuous and categorical variables between groups were compared by independent samples *t* test and chi‐square test, respectively. The significant variables in the univariable analysis were selected for multivariable analysis. Time to the CV and overall mortality events and covariates of risk factors were modeled using the Cox proportional hazards model with forward selection. Receiver operating characteristic curves are used for comparing different models for prediction of CV and overall mortality. The test with the higher area under curve (AUC) is considered better. The incremental value of ePWV and baPWV over basic model to predict CV and overall mortality was studied by calculating the improvement in global chi‐square value. Discriminatory ability was evaluated by calculating the net reclassification improvement (NRI). All tests were 2‐sided and the level of significance was established as *p* < .05.

## 3 RESULTS

Among the 881 subjects, mean age was 61 ± 13 years. CV and overall mortality data were collected up to December 2018. Mortality data were obtained from the Collaboration Center of Health Information Application (CCHIA), Ministry of Health and Welfare, Executive Yuan, Taiwan. The follow‐up period to mortality events was 94 (25th–75th percentile: 87–101) months in all patients. Mortality events were documented during the follow‐up period, including CV mortality (*n* = 66) and overall mortality (*n* = 184).

Table 1 compares the clinical characteristics between patients with ePWV below and above the median (10.3 m/s). Compared to patients with ePWV below the median, patients with ePWV above the median had an older age, more female gender, higher prevalence of diabetes and hypertension, lower prevalence of smoking, higher systolic blood pressure, higher ePWV and baPWV, and higher percentage of aspirin, and calcium channel blocker use.

**TABLE 1:** Comparison of clinical characteristics between patients with ePWV below and above the median (10.3 m/s)

Baseline characteristics	ePWV below the median	ePWV above the median	*p* value
Number	460	421	
Age (years)	52 ± 10	71 ± 9	<.001
Male gender (%)	62.2%	48.7%	<.001
Smoking (%)	20.2%	9.0%	<.001
Diabetes (%)	21.3%	32.5%	<.001
Hypertension (%)	62.1%	80.0%	<.001
Coronary artery disease (%)	16.7%	17.1%	.928
Heart failure (%)	6.1%	7.1%	.587
SBP (mmHg)	126 ± 16	145 ± 20	<.001
Total cholesterol	193 ± 43	188 ± 37	.063
Heart rate (min^−1^)	70 ± 12	69 ± 12	.657
PWV			
ePWV (m/s)	8.5 ± 1.1	12.2 ± 1.5	<.001
baPWV (m/s)	15.1 ± 2.5	20.2 ± 4.5	<.001
Medication			
Aspirin	27.9%	34.8%	.029
β‐blockers	40.8%	39.0%	.629
CCBs	32.5%	43.8%	.001
ACEIs/ARBs	52.7%	57.6%	.154
Diuretics	26.9%	30.5%	.232

Abbreviations: ACEI, angiotensin‐converting enzyme inhibitor; ARB, angiotensin II receptor blocker; baPWV, brachial‐ankle pulse wave velocity; CCB, calcium channel blocker; ePWV, estimated pulse wave velocity; PWV, pulse wave velocity; SBP, systolic blood pressure.

The univariable analysis of Cox proportional hazards model found increased CV mortality was associated old age, the presence of diabetes, coronary artery disease, and heart failure, high systolic blood pressure, high heart rate, diuretic use, high ePWV, and high baPWV, and increased overall mortality was associated with old age, the presence of diabetes, coronary artery disease, and heart failure, high systolic blood pressure, low total cholesterol, high heart rate, diuretic use, high ePWV, and high baPWV. In direct comparison of this univariable analysis, ePWV had a better predictive value for CV mortality (chi‐square value: 47.00 versus 38.39, *p* = .003) but similar predictive value for overall mortality (chi‐square value: 134.18 versus 130.58, *p* = .058) as baPWV.

Table 2 shows the predictors of CV mortality using Cox proportional hazards model in the multivariable analysis. After adjusting significant variables in the univariable analysis, including age, diabetes, coronary artery disease, heart failure, systolic blood pressure, heart rate, diuretic use, both ePWV (hazard ratio [HR] = 2.321; 95% confidence interval [CI]: 1.800–2.994; *p* < .001) and baPWV (HR = 1.385; 95% CI: 1.102–1.742; *p* = .005) were significantly associated with CV mortality.

**TABLE 2:** Predictors of CV mortality using Cox proportional hazards model (multivariable analysis with forward selection)

Parameter	CV mortality (PWV: using ePWV)		CV mortality (PWV: using baPWV)	
	HR (95% CI)	*p*	HR (95% CI)	*p*
Age (+13.71 year)	–	–	2.186 (1.588–3.011)	<.001
Diabetes (yes vs. no)	2.070 (1.258–3.405)	.004	1.988 (1.211–3.262)	.007
Coronary artery disease	1.810 (1.012–3.239)	.046	–	–
Heart failure	7.343 (4.244–12.707)	<.001	7.526 (4.351–13.018)	<.001
SBP (+20.80 mmHg)	–	–	–	–
Heart rate (+12.33 beat/min)	–	–	–	–
Diuretic use	–	–	–	–
PWV*	2.321 (1.800–2.994)	<.001	1.385 (1.102–1.742)	.005

Note

The HRs of continuous variables were calculated as a standard deviation change.

Age, diabetes, SBP, heart rate, diuretic use, and PWV were significant variables in the univariable analysis. Covariates in the multivariable model included the above significant variables in the univariable analysis. *Standard deviation for ePWV: +2.36 m/s; standard deviation for baPWV: +4.66 m/s.

Abbreviations: CI, confidence interval; HR, hazard ratio; other abbreviations as in Table 1.

Table 3 shows the predictors of overall mortality using Cox proportional hazards model in the multivariable analysis. After adjusting significant variables in the univariable analysis, including age, diabetes, coronary artery disease, heart failure, systolic blood pressure, total cholesterol, heart rate, diuretic use, both ePWV (HR = 1.640; 95% CI: 1.162–2.315; *p* = .005) and baPWV (HR = 1.570; 95% CI: 1.340–1.839; *p* < .001) were still significantly associated with overall mortality.

**TABLE 3:** Predictors of overall mortality using Cox proportional hazards model (multivariable analysis with forward selection)

Parameter	Overall mortality (using ePWV)		Overall mortality (using baPWV)	
	HR (95% CI)	*p*	HR (95% CI)	*p*
Age (+13.71 year)	1.672 (1.129–2.478)	.01	2.250 (1.791–2.826)	<.001
Diabetes (yes vs. no)	1.943 (1.382–2.733)	<.001	1.802 (1.279–2.539)	.001
SBP (+20.80 mmHg)	–	–	–	–
Coronary artery disease	–	–		
Heart failure	3.660 (2.316–5.784)	<.001	3.802 (2.405–6.012)	<.001
Total cholesterol (+40.77 mg/dl)	0.742 (0.613–0.899)	.002	0.757 (0.628–0.912)	.03
Heart rate (+12.33 beat/min)	–	–	–	–
Diuretic use	–	–	–	–
PWV*	1.640 (1.162–2.315)	.005	1.570 (1.340–1.839)	<.001

Note

The HRs of continuous variables were calculated as a standard deviation change.

Age, diabetes, SBP, total cholesterol, heart rate, diuretic use, and PWV were significant variables in the univariable analysis. Covariates in the multivariable model included the above significant variables in the univariable analysis. *Standard deviation for ePWV: +2.36 m/s; standard deviation for baPWV: +4.66 m/s.

Abbreviations: CI, confidence interval; HR, hazard ratio; other abbreviations as in Table 1.

Table 4 shows the comparison of AUC between FRS, ePWV, and baPWV for prediction of CV and overall mortality. The unadjusted AUC between FRS, ePWV, and baPWV for prediction of CV mortality was 0.681, 0.734, and 0.690, respectively. We found that there was a significant difference of AUC between ePWV and FRS (*p* = .044), but non‐significant difference between baPWV and FRS (*p* = .782). In addition, the unadjusted AUC between FRS, ePWV, and baPWV for prediction of overall mortality were 0.703, 0.766, and 0.722, respectively. We found that there was also a significant difference of AUC between ePWV versus FRS (*p* < .001), but non‐significant difference between baPWV and FRS (*p* = .367).

**TABLE 4:** Comparison of unadjusted AUC between FRS, ePWV, and baPWV for prediction of CV and overall mortality

	Comparison of AUC	*p* value
CV mortality		
ePWV vs. FRS	0.734 vs. 0.681	.044
baPWV vs FRS	0.690 vs. 0.681	.782
Overall mortality		
ePWV vs. FRS	0.766 vs. 0.703	<.001
baPWV vs FRS	0.722 vs. 0.703	.367

Abbreviations: AUC, area under curve; baPWV, brachial‐ankle pulse wave velocity; ePWV, estimated pulse wave velocity; FRS, Framingham risk score.

**FIGURE 1:**
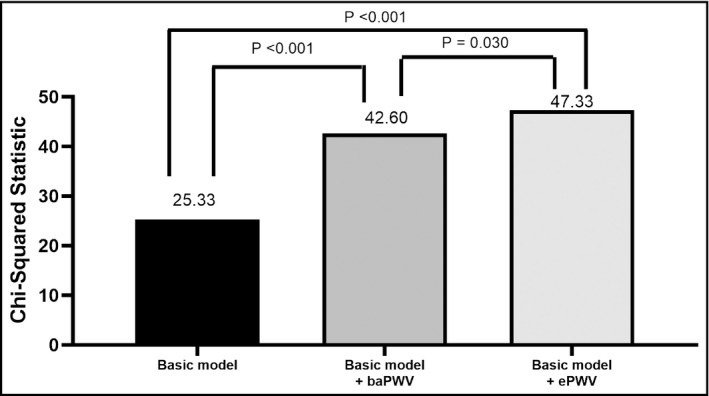
Direct comparison among basic model, basic model + brachial‐ankle pulse wave velocity (baPWV), and basic model + estimated pulse wave velocity (ePWV) for cardiovascular mortality prediction in multivariable analysis. Framingham risk score was used as the basic model

Figure 1 shows the Nested Cox model for CV mortality prediction. We used FRS as the basic model. The basic model could significantly predict CV mortality (chi‐square value, 25.33, *p* < .001). We further added baPWV and ePWV into the basic model. Both basic model + ePWV and basic model + baPWV could provide an extra benefit in prediction of CV mortality than basic model (*p* < .001). In direct comparison between basic model + baPWV and basic model + ePWV, the basic model + ePWV had a better predictive value for CV mortality (*p* = .030).

**FIGURE 2:**
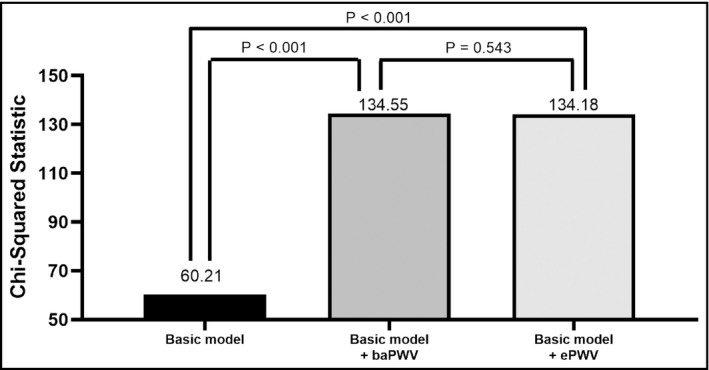
Direct comparison among basic model, basic model + brachial‐ankle pulse wave velocity (baPWV), and basic model + estimated pulse wave velocity (ePWV) for overall mortality prediction in multivariable analysis. Framingham risk score was used as the basic model

Figure 2 shows the Nested Cox model for overall mortality prediction. The basic model could significantly predict overall mortality (Chi‐square vale, 60.21, *p* < .001). We further added baPWV and ePWV into the basic model. Both basic model + baPWV and basic model + ePWV could provide an extra benefit in prediction of overall mortality than basic model (*p* < .001). In direct comparison between basic model + baPWV and basic model + ePWV, the basic model + ePWV had similar predictive value for overall mortality as basic model + baPWV (*p* = .543).

We also performed NRI to evaluate the discriminatory ability after adding ePWV and baPWV into basic model including FRS for prediction of CV and overall mortality. The results were shown in Table 5. We found that NRI improved significantly after adding ePWV and baPWV into FRS for prediction of CV (*p* ≤ .02) and overall mortality (*P* < .001).

**TABLE 5:** Net reclassification improvement analysis for CV and overall mortality prediction after adding ePWV and baPWV into FRS model

Model	Net reclassification improvement	*p* value
CV mortality		
FRS + ePWV vs. FRS	0.37 (0.15–0.59)	.001
FRS + baPWV vs FRS	0.48 (0.04–0.48)	.02
Overall mortality		
FRS + ePWV vs. FRS	0.47 (0.33–0.62)	<.001
FRS + baPWV vs FRS	0.34 (0.19–0.48)	<.001

Abbreviations: AUC, area under curve; baPWV, brachial‐ankle pulse wave velocity; ePWV, estimated pulse wave velocity; FRS, Framingham risk score.

## 4 DISCUSSION

This study aimed to evaluate the ability of ePWV in predicting CV and overall mortality and compare the predictive value of CV and overall mortality between ePWV and baPWV. There are several major findings in the present study. First, both increased ePWV and baPWV were associated with increased CV and overall mortality in the univariable and multivariable analyses. Second, ePWV had better predictive value than FRS for prediction of CV and overall mortality. However, baPWV did not. Third, in direct comparison of univariable and multivariable analysis, ePWV had a better additive predictive value for CV mortality than baPWV but similar predictive value for overall mortality as baPWV.

The ePWV calculated by equations using age and MBP has shown to be a reliable parameter of arterial stiffness as measured carotid‐femoral PWV.^11^ Greve et al reported that ePWV could predict composite CV endpoints of CV death, nonfatal myocardial infarction, nonfatal stroke, and hospitalization for ischemic heart disease independently of Systematic COronary Risk Evaluation (SCORE) or FRS as well as carotid‐femoral PWV.^11^ In addition, in the secondary analysis of SPRINT study, Vlachopoulos et al also showed that ePWV could predict outcomes independent of the FRS and could be used to gauge the effect of treatment of hypertension.^14^ In the present study, we consistently demonstrated that high ePWV was associated with increased CV and overall mortality.

Increased PWV, which reflects increased arterial stiffness, was reported to be an independent predictor of CV outcomes and prognosis.^1^, ^2^, ^3^, ^4^, ^5^, ^6^, ^26^ PWV was also associated with atherosclerosis,^27^, ^28^ left ventricular diastolic dysfunction,^29^, ^30^ left ventricular mass index, and left ventricular hypertrophy.^31^, ^32^, ^33^, ^34^, ^35^ Although several parameters can be used to measure arterial stiffness, the gold standard non‐invasive method was carotid‐femoral PWV,^18^ which was reported to directly reflect aortic PWV.^36^, ^37^ In comparison, baPWV was a composite measure of several arterial segments, and some of these segments would be prone to arteriosclerosis (brachial and distal arteries). In Hatsuda's study, they found in patients with type 2 diabetes mellitus, central arterial stiffness played a more important role in the development of ischemic heart disease than peripheral arterial stiffness.^38^ The ePWV was an estimate of central arterial stiffness,^11^ but baPWV was a mixture of central and peripheral arterial stiffness. Central arterial stiffness might have a more important contribution in the development of CV disease. Therefore, our present study similarly showed ePWV had a superior predictive valve for CV mortality than baPWV both in the univariable and multivariable analyses.

Choo et al found in healthy subjects, carotid‐femoral PWV displayed a strong correlation with central heart‐femoral PWV, whereas baPWV displayed a moderate correlation with both central heart‐femoral PWV and peripheral femoral‐ankle PWV.^39^ In the present study, both ePWV and baPWV were significant predictors of overall mortality in the univariable and multivariable analyses. In addition, baPWV also had similar predictive value for overall mortality as ePWV in the univariable (*p* = .058) and multivariable analysis (*p* = .541). The underlying mechanism of this finding was unknown. However, both central and peripheral arterial stiffness should have a certain role in survival predication. BaPWV, a mixture of central and peripheral arterial stiffness, might also exhibit a good predictive value for long‐term overall mortality as ePWV, an estimated measure of central arterial stiffness.

### 4.1 Study limitations

There were some limitations to this study. First, the sample size of our study was not very large, but the follow‐up period was relatively long, up to 105 months. Second, the majority of our patients were treated with CV drugs. For ethical reasons, we did not withdraw these medications. Hence, we could not exclude the influence of CV drugs on our study. However, we adjusted the associated usage of CV drugs in the multivariable analysis. Third, our study was aimed to evaluate the mortality events, so nonfatal events were not studied.

## 5 CONCLUSIONS

Our study was the first one to compare ePWV and baPWV for prediction of long‐term CV and overall mortality. We found both ePWV and baPWV were independent predictors for long‐term CV and overall mortality in univariable and multivariable analysis. ePWV had better predictive value than FRS for CV and overall mortality prediction but baPWV did not. In addition, ePWV had a better additive predictive value for CV mortality than baPWV and similar predictive value for overall mortality as baPWV. Therefore, ePWV obtained without equipment deserved to be calculated for overall mortality prediction and better CV survival prediction.

## CONFLICT OF INTEREST

The authors have declared no competing interest exists.

## AUTHOR CONTRIBUTIONS

Conceptualization, Po‐Chao Hsu, Wen‐Hsien Lee, Cheng‐An Chiu; Data curation, Wei‐Chung Tsai and Ying‐Chih Chen; Formal analysis, Hsueh‐Wei Yen and Wei‐Chung Tsai; Investigation, Po‐Chao Hsu, Chun‐Yuan Chu and Tsung‐Hsien Lin; Methodology, Wen‐Chol Voon, Wen‐Ter Lai and Sheng‐Hsiung Sheu; Supervision, Wen‐Chol Voon, Wen‐Ter Lai, Sheng‐Hsiung Sheu and Ho‐Ming Su; Validation, Wen‐Ter Lai, Sheng‐Hsiung Sheu and Ho‐Ming Su; Visualization, Wen‐Ter Lai, Sheng‐Hsiung Sheu, Ho‐Ming Su, and Cheng‐An Chiu; Writing – original draft, Po‐Chao Hsu and Wen‐Hsien Lee; Writing – review & editing, Ho‐Ming Su and Cheng‐An Chiu.
